# *Hif1α *down-regulation is associated with transposition of great arteries in mice treated with a retinoic acid antagonist

**DOI:** 10.1186/1471-2164-11-497

**Published:** 2010-09-16

**Authors:** Francesca Amati, Laura Diano, Luisa Campagnolo, Lucia Vecchione, Daria Cipollone, Susana Bueno, Gianluca Prosperini, Alessandro Desideri, Gregorio Siracusa, Giovanni Chillemi, Bruno Marino, Giuseppe Novelli

**Affiliations:** 1 Department of Biopathology, Tor Vergata University, Via Montpellier 1, 00133, Rome, Italy; 2 Interdisciplinary Centre for Bioinformatics and Biostatistics, Tor Vergata University, Via Montpellier 1, 00133, Rome, Italy; 3 Department of Public Health and Cell Biology, Tor Vergata University, Via Montpellier 1, 00133, Rome, Italy; 4 Department of Pediatrics, La Sapienza University, Viale Regina Elena 324, 00161 Rome, Italy; 5 CASPUR, Consortium for Supercomputing Applications, Via dei Tizii 6, 00185, Rome, Italy; 6 Deptartment of Biology, Tor Vergata University, Via della Ricerca Scientifica 1, 00133, Rome, Italy; 7 St. Peter Fatebenefratelli Hospital, Via Cassia 600, 00189, Rome, Italy; 8 Department of Internal Medicine, University of Arkansas for Medical Sciences and Central Arkansas, Veterans Healthcare System, Little Rock, AR, USA

## Abstract

**Background:**

Congenital heart defect (CHD) account for 25% of all human congenital abnormalities. However, very few CHD-causing genes have been identified so far. A promising approach for the identification of essential cardiac regulators whose mutations may be linked to human CHD, is the molecular and genetic analysis of heart development. With the use of a triple retinoic acid competitive antagonist (BMS189453) we previously developed a mouse model of congenital heart defects (81%), thymic abnormalities (98%) and neural tube defects (20%). D-TGA (D-transposition of great arteries) was the most prevalent cardiac defect observed (61%). Recently we were able to partially rescue this abnormal phenotype (CHD were reduced to 64.8%, p = 0.05), by oral administration of folic acid (FA). Now we have performed a microarray analysis in our mouse models to discover genes/transcripts potentially implicated in the pathogenesis of this CHD.

**Results:**

We analysed mouse embryos (8.5 dpc) treated with BMS189453 alone and with BMS189453 plus folic acid (FA) by microarray and qRT-PCR. By selecting a fold change (FC) ≥ ± 1.5, we detected 447 genes that were differentially expressed in BMS-treated embryos vs. untreated control embryos, while 239 genes were differentially expressed in BMS-treated embryos whose mothers had also received FA supplementation vs. BMS-treated embryos. On the basis of microarray and qRT-PCR results, we further analysed the *Hif1α *gene. In fact *Hif1α *is down-regulated in BMS-treated embryos vs. untreated controls (FC_micro _= -1.79; FC_qRT-PCR _= -1.76; p = 0.005) and its expression level is increased in BMS+FA-treated embryos compared to BMS-treated embryos (FC_micro _= +1.17; FC_qRT-PCR _= +1.28: p = 0.005). Immunofluorescence experiments confirmed the under-expression of Hif1α protein in BMS-treated embryos compared to untreated and BMS+FA-treated embryos and, moreover, we demonstrated that at 8.5 dpc, Hif1α is mainly expressed in the embryo heart region.

**Conclusions:**

We propose that Hif1α down-regulation in response to blocking retinoic acid binding may contribute to the development of cardiac defects in mouse newborns. In line with our hypothesis, when Hif1α expression level is restored (by supplementation of folic acid), a decrement of CHD is found. To the best of our knowledge, this is the first report that links retinoic acid metabolism to Hif1α regulation and the development of D-TGA.

## Background

Congenital heart defects affect 1-2% of newborns and are the leading cause of death in infants under one year of age [1]. While the overwhelming majority of congenital heart malformations do not segregate in Mendelian ratios, they do show familial aggregation, which suggests that genetic factors play a role in their development [2,3]. Despite this, a limited number of CHD-causing genes have been identified so far [4].

Isolated D-Transposition of great arteries (D-TGA, OMIM 608808) accounts for 5% of all congenital heart diseases [5]. Its incidence is estimated at 1 in 3,500-5,000 live births [6]. Most D-TGA cases are sporadic, but familial cases have also been reported [7]. A discrete number of causing genes have been identified so far (*ZIC3*, *CFC1, THRAP2, GDF1*, *NODAL*), but their mutation explains only a minority of cases [8-13]. Interestingly, many of these genes participate in embryonic left-right axis patterning [14]. Moreover, D-TGA has been observed to be frequently related to laterality defects (failure to establish a normal left-right asymmetry during embryonic development), in particular, in patients with asplenia/right isomerism. Conversely, one of the most prevalent types of CHD in lateralisation defects is D-TGA [15].

Transcriptome analysis using DNA microarrays has become a standard approach for investigating the molecular basis of human disease in both clinical and experimental settings, as the pattern of transcriptional deregulation may provide insights into the cause of abnormal phenotypes, including congenital defects [16-20].

In the present study we have analysed the transcriptome of mouse embryos whose development was dramatically altered by temporarily blocking retinoic acid signalling and of embryos in which the abnormal developmental phenotype was rescued by a concomitant supplementation with folic acid [21,22].

We previously administered to pregnant mice BMS189453, a synthetic retinoic acid (RA) antagonist having good (82-98%) oral bioavailability in rats and monkeys [21]. BMS189453 binds, but does not activate, the α, β, and γ retinoid receptors [23]. Oral administration of BMS189453 to pregnant mice twice, at 7.25/7.75 dpc (days post coitum), induces cardiac defects (81%), thymic abnormalities (98%) and neural tube defects (20%) at birth [21]. Concomitant oral supplementation with FA, during pregnancy, partially rescues this abnormal phenotype [22]. In particular, FA reduces congenital heart diseases from 81.3% to 64.8%, neural tube defects from 20.3% to 3.7% and thymic abnormalities from 98.4% to 27.8%, restoring a normal number of differentiated thymic cells [22].

To better identify genes/transcripts involved in the pathogenesis of the congenital defects observed in our mouse models, we performed a global microarray analysis on embryos. To identify the best developmental stage for microarray screening, we first analysed the gene expression pattern of *Rarα*, a retinoic acid responsive gene in mouse embryos, at 8.5, 9.5 and 11.5 dpc. At 8.5 dpc, all embryos analysed showed down-regulation of *Rarα *mRNA, compared to only 70% of the embryos at 9.5 dpc and 50% of embryos at 11.5 dpc (data not shown). Thus, we thus decided to analyse the gene expression pattern in 8.5 dpc embryos.

The data presented in this paper reveal that changes in the expression level of *Hif1α *(hypoxia-inducible factor 1 alpha subunit) during mouse embryogenesis are associated with CHD observed in our mouse models.

## Methods

### BMS-189453 and folic acid treatment protocol and embryo recovery

Outbred CD1, Swiss mice (Charles River, Calco, Italy) were housed and mated under standard laboratory conditions that conform to the Guide for the Care and Use of Laboratory Animals published by the US National Institutes of Health (NIH Publication No. 85-23, revised 1996); an Italian ministerial authorization (DL 116/92) was obtained to carry out the experimental treatment protocols. During the experimental period, all animals had free access to water and a conventional laboratory diet (Standard diet n.48) until sacrifice. Room temperature was kept at 21 ± 2°C and 12 h of light was automatically alternated with 12 h of darkness.

Pregnant mice were randomly divided into three experimental groups of 6 mice each (untreated controls, BMS-treated and BMS+FA-treated group). Administration of BMS189453 (Bristol-Myers-Squibb, Princeton, NJ, USA) both alone and together with folic acid to pregnant mice was performed as described [21,22].

Mouse embryos were collected at 8.5 dpc, and placentas were removed from embryonic tissue. Three to five embryos were pooled within each litter, and stored in RNAlater (Ambion) until RNA extraction.

### RNA extraction, preparation and hybridisation of cDNA probe

Total RNA was extracted and purified using TriZol reagent (Invitrogen), and its quality and quantity was assessed using a Nanodrop spectrophotometer (Thermo Scientific) and agarose gel electrophoresis.

Synthesis of the labelled first strand cDNA was conducted according to manufacturer's instructions (Superscript Indirect cDNA labelling system, Invitrogen, USA) with starting material of 10 μg of total RNA. Briefly, the amino-allyl labelled dNTP mix was added to the reaction to generate amino-allyl labelled second strand cDNA. Following the hydrolysis reaction, single-stranded cDNA probes were purified using a Purification Module (Invitrogen). Probe mixtures where then evaporated in a vacuum centrifuge, and the cDNA pellet resuspended in 3 μL of water. The dye-coupling reactions were performed by mixing the cDNA samples with AlexaFluor Dyes 555 or 647 and were incubated for overnight in the dark. The reactions were purified with a Purification Module (Invitrogen) to remove the unincorporated/quenched dyes. After the purification, samples were combined for hybridisation. The labelled cDNAs were co-hybridised to slides in duplicate with one dye swap.

Microarray slides contained approximately 33.000 oligonucleotides corresponding to the whole mouse genome (AECOM, USA, http://microarray1k.aecom.yu.edu/).

### Image Analysis and Processing

Each slide was scanned on the GenePix 4000B Microarray Scanner at the optimal wavelength for the Alexa555 (F532) and Alexa647 (F635) dyes.

The spots were automatically segmented; total intensities as well as the fluorescence ratios of the two dyes for each spot were then calculated. The spots were flagged when they exhibited poor hybridisation signals and when they were saturated (F635 or F532 median = 65535). Spots with a signal to background ratio below 1.5 were filtered together with flagged spots. We decided to subtract the local spot background signal from the foreground signal depending on the correlation of foreground to background intensity ratios, as in the method suggested by Scharpf e al. [24].

We removed systematic bias in the data by applying lowess (smoother span 2/3) [25] and dye-swap normalisations to have the least possible information loss. Dye-swap normalisation makes use of reverse labelling in the two microarray replicates directly [26]. To establish the significance of the observed regulation for each gene, we used a one sample t-test and corrected the p-value for multiple comparisons controlling the false discovery rate [27]. We transformed FC < 1 in FC* = -1/FC. Finally, only genes with a satisfactory effect (Fold Change, FC≥ ± 1.5) were considered.

### Validation of relative gene expression by real-time RT-PCR

Two μg of total RNA was reverse-transcribed into cDNA according to manufacturer's instructions (High-Capacity cDNA Archive Kit, Applied Biosystems, Foster City, CA USA). The expression levels of the selected genes and an internal reference (*ribosomal 18S*) were measured by multiplex PCR using Assay-on-Demand™ gene expression products (Applied Biosystems, Foster City, CA USA) labelled respectively with 6 carboxyfluorescein (FAM) (selected genes) and VIC (internal reference) (Applied Biosystems). We analysed the following genes: *Hif1α *(Mm00468869_m1), *Mospd3 *(Mm00551672_g1), *Mgp *(Mm00485009_m1), *Sat1 *(Mm01198850_m1), *Canx *(Mm00500330_m1), *Tfpi *(Mm00803534_m1), *Rarα *(Mm00436264_m1), *Cited2 *(Mm00516121_m1). We performed PCRs using the TaqMan Universal PCR Master Mix and the ABI PRISM 7000 Sequence Detection System. All samples were run in triplicate and average values were calculated. Each qRT-PCR experiment was repeated at least twice.

Relative quantification of gene expression among each sample was achieved by normalisation against ribosomal 18S endogenous control using the ΔΔCt method of quantification. The relative amount of mRNA was calculated as 2-^ΔΔCt^. Data are mean ± standard error of the mean (SEM). A one-way analysis of variance (ANOVA) and t-test were applied to look for significant differences between experimental conditions for each candidate gene. A p value < 0.05 was considered statistically significant. Calculations were performed using the 2.9.1 version of R software (http://www.r-project.org/).

### Immunofluorescence analysis

Embryos at 8.5 dpc were fixed in 4% paraformaldehyde overnight at 4°C and processed for paraffin embedding and sectioning, following standard procedures. After blocking non-specific antibody binding in 10% normal donkey serum for 1 h at room temperature, 10-μm sections were incubated with antibodies against Hif1α (10 μg/ml; Novus Biologicals) and α-actinin (2 μg/ml; Abcam) overnight at 4°C. After several washes in PBS, sections were incubated with donkey anti-rabbit AlexaFluor488 or donkey anti-rat Alexa568 (2 μg/ml; Invitrogen), depending on the primary antibody used. Nuclei were counterstained with 0.5 μg/ml Hoechst 33258 in PBS. Slides were mounted with Möwiol and fluorescent images were taken with a Zeiss Axioplan2 microscope.

### *In silico *search for RARE elements

We searched for retinoic acid response elements (RARE) in the aligned human and murine 5-Kb region upstream of the transcription start site of *Hif1α *gene. RAREs consist of direct repeats (DR) of two nucleotide motifs PuG(G/T)TCA usually separated by 5, 2 or 1 intervening nucleotides. However, few other forms of RARE have been characterised, with different half-site consensus sequences or with diverse spacer length [28,29].

The 5-kb human and murine regions were obtained from the UCSC Genome Bioinformatics Site (http://genome.ucsc.edu) and potential RARE sequences were searched using in-house written codes.

## Results

### Transcriptome analysis of BMS189453-treated embryos

We previously reported that BMS189453 oral administration to pregnant mice was responsible for congenital defects in newborns [21], while a concomitant FA supplementation during pregnancy partially rescued the abnormal phenotype [22].

In this study, we first analysed the transcriptome dysregulation induced by altering RA binding in 8.5 dpc mouse embryos using a comparative microarray approach. The expression data of all of the experiments are available as a specific GEO Sample record with accession number GSE19012 (http://www.ncbi.nlm.nih.gov/geo/).

**Figure 1:**
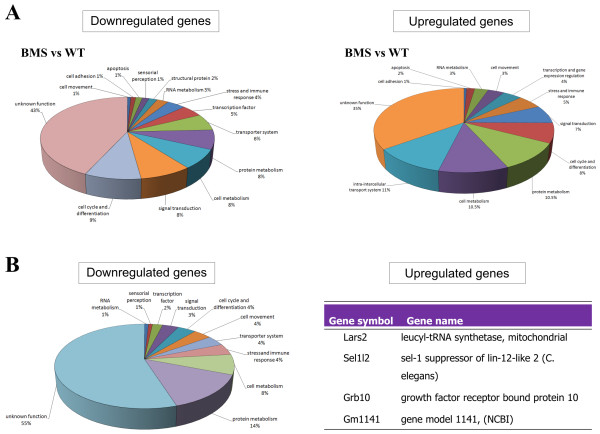
**Graphic illustration of GO classification of up- and down-regulated genes (FC≥ ± 1.5) in BMS-treated (A) and BMS+FA-treated embryos (B)**.

After data normalisation 447 genes were differentially expressed in BMS-treated embryos vs. untreated control embryos (Additional file 1: Table S1). Among these genes, 276 were down-regulated while 171 were up-regulated. According to GO classification, the down-regulated genes code for proteins involved in the cell cycle and differentiation (9.0%), signal transduction (8.3%), cellular metabolism (7.6%) and protein metabolism (7.6%), while the up-regulated genes were mainly involved in transport (11.1%), cellular metabolism (10.5%) and protein metabolism (10.5%) (Figure 1A).

### Comparison of the gene expression profiles of BMS-treated and BMS+FA-treated embryos

Next, we analysed the gene expression pattern of embryos exposed to BMS-189453 only with that of embryos exposed to FA supplementation as well. In BMS-treated embryos whose mothers had also received FA supplementation vs. BMS-treated ones, 239 genes were differentially expressed after data normalisation. Interestingly, among these 239 genes, 235 were down-regulated, while only four were up-regulated (Additional file 2: Table S2). According to GO classification, the down-regulated genes were mainly involved in protein metabolism (14%) and cellular metabolism (8%) while the four up-regulated genes code for proteins involved in signal transduction (Figure 1B).

We then compared the 447 differentially expressed genes from the first experiment (BMS-treated embryos vs. untreated ones) with those (239) of the second experiment (BMS+FA-treated vs. BMS-treated-embryos). A total of 140 genes were commonly expressed in both the experiments. Furthermore, we analysed the potential involvement of these 140 genes in the development of the heart region by a combination of extensive database mining and *in silico *analysis of gene expression (GenePaint database). We identified 44 genes that might be potentially relevant to mouse cardiogenesis (Table 1, Table 2).

**Table 1:** List of the 26 genes that have similar expression patterns in BMS- and BM+FA-treated embryos.

Gene symbol	Gene name	FC BMS/untreated controls	FC BMS+FA/BMS	GO classification
*Tmem176b*	transmembrane protein 176B	-2.58	-1.08	cellular differentiation
*Pcsk5*	proprotein convertase subtilisin/kexin type 5	-2.06	-1.08	cardiac development
*Ankrd13c*	ankyrin repeat domain 13c	-1.58	-1.06	unknown biological process
*Slc7a15*	solute carrier family 7 member 15	-2.23	-1.12	amino acid transporter
***Canx***	**calnexin**	**-1.51**	**-1.07**	**protein folding**
*Rab11b*	RAB11B, member RAS oncogene family	-1.60	-1.31	signal transduction
*Ralbp1*	ralA binding protein 1	-1.53	-1.12	signal transduction
***Sat1***	**spermidine/spermine N1-acetyl transferase1**	**-1.62**	**-2.28**	**regulation of cell proliferation**
*Fn1*	fibronectin 1	-1.58	-1.24	cell-matrix adhesion
*Pcbp1*	poly(rC) binding protein 1	-1.57	-1.59	mRNA processing
*Naca*	nascent polypeptide-associated complex alpha polypeptide	-1.64	-1.70	regulation of transcription
*Rpl39*	ribosomal protein L39	1.71	-2.10	translation
***Mospd3***	**motile sperm domain containing 3**	**-1.92**	**-1.24**	**cardiac development**
*Eif4a1*	eukaryotic translation initiation factor 4A1	-1.56	-1.19	translation initiation factor activity
*Rps15a*	ribosomal protein S15a	-1.77	-2.54	translation
*Gpr82*	G protein-coupled receptor 82	-1.59	-1.21	G-protein coupled receptor protein signalling pathway
*Eif5a*	eukaryotic translation initiation factor 5A	-1.81	-1.36	translation initiation factor activity
*EG23* *4159*	predicted gene, EG234159	-1.50	-1.01	unknown
*LOC63* *3468*	similar to H3 histone, family 3B	-1.77	-1.27	unknown
XM_920276	/	-1.58	-3.10	unknown
XM_921367	/	-4.86	-1.00	unknown
*OTTMUSG* *0000000* *4999*	predicted gene, OTTMUSG 00000004999	-1.64	-2.14	unknown
XM_980000	/	-1.96	-2.42	unknown
XM_990335	/	-4.16	-1.04	unknown
XR_002657	/	-2.11	-6.27	unknown

Genes in bold have been analysed by qRT-PCR

**Table 2:** List of the 18 genes that have different expression patterns in BMS- and BMS+FA-treated embryos.

Gene symbol	Gene name	FC BMS/untreated controls	FC BMS+FA/BMS	GO classification
***Tfpi***	**tissue factor pathway inhibitor**	**-1.79**	**+1.18**	**coagulation**
***Mgp***	**Matrix Gla protein**	**+1.73**	**-2. 77**	**cell differentiation**
*Rragb*	Ras-related GTP binding B	-1.589	+1.11	signal transduction
*Hs3st6*	heparan sulphate (glucosamine) 3-O-sulfotransferase 6	-1.55	+1.40	heparan sulphate synthesis
*B2m*	beta-2 microglobulin	-1.71	+1.28	immune response
***Hif1α***	**hypoxia inducible factor 1, alpha subunit**	**-1.79**	**+1.17**	**cardiac looping**
*Rpn2*	ribophorin II	+1.78	-1.59	protein glycosylation
*Rhoj*	ras homolog gene family, member J	-1.67	+1.30	signal transduction
*Spcs2*	signal peptidase complex subunit 2 homolog (S. cerevisiae)	+1.70	1.38	signal peptide processing
*Atp6v1b1*	ATPase, H+ transporting, lysosomal V1 subunit B1	+2.08	1.01	proton transport
*Lmbrd2*	LMBR1 domain containing 2	-1.88	+1.16	unknown biological process
*Rnf217*	ring finger protein 217	+2.05	-1.26	unknown biological process
*Vmn2r52*	vomeronasal 2, receptor 52	-1.67	+1.26	transmembrane receptor
*D14Abb1e*	DNA segment, Chr 14, Abbott 1 expressed	-1.70	+1.38	unknown
XM_981869		+1.78	-1.75	unknown
XM_991521		+1.55	-1.24	unknown
XM_994830		-2.37	+1.13	unknown
NM_133970		-1.51	+1.40	unknown

Genes in bold have been analysed by qRT-PCR.

### qRT-PCR assays

Additionally, to confirm the expression pattern revealed by our microarray assays, we analysed six differentially expressed genes (*Mgp*, *Tfpi, Hif1α*, *Mospd3*, *Canx*, *Sat1*) by qRT-PCR (Table 1 and Table 2, in bold). We selected genes that had a similar expression pattern (up-regulated or down-regulated) in both embryos groups (*Canx*, *Sat1*, *Mospd3*; Table 1) and genes that showed a different expression pattern in both embryos groups (i.e. if down-regulated in the BMS-treated group, they were up-regulated in the BMS+FA-treated group, and vice-versa) (*Mgp*, *Tfpi*, *Hif1α*; Table 2). The housekeeping gene *18SRNA *was used as an internal control.

**Figure 2:**
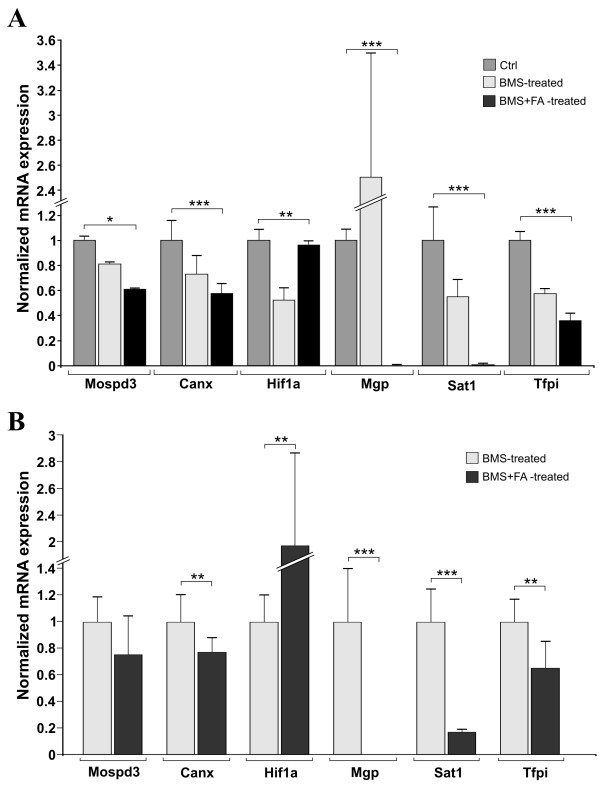
**Quantitative RT-PCR gene expression analysis in BMS treated and BMS+FA-treated embryos**. Fold change in gene expression was analysed by the 2-^ΔΔCt ^(see methods for details) of six selected genes in untreated controls, BMS- and BMS+FA-treated embryos (A) and in BMS+FA vs. BMS-treated embryos (B). Data are mean ± S.E.M., n = 6; * p < 0.05, ** p < 0.01, *** p < 0.0001.

The mRNA expression values of these selected genes in untreated controls, and BMS- and BMS+FA-treated embryos are represented in Figure 2A. We also evaluated the expression level of each gene in BMS+FA- vs. BMS-treated embryos (Figure 2B). As indicated in Figure 2, the qRT-PCR results are in very good agreement with the expression patterns of the microarray experiments (Table 1 and Table 2).

### *Hif1α *mRNA and protein are down-regulated in BMS-treated embryos, but their expression is recovered by supplementation with folic acid

Among the six genes analysed by qRT-PCR, *Hif1α *expression pattern was noteworthy; in fact, *Hif1α *was down-regulated in BMS-treated embryos (FC_micro _= -1.79; FC_qRT-PCR _= -1.76, p = 0.005 Table 1, Figure 2A and 2B) but its mRNA level increased by about 73% in BMS+FA-treated embryos (FC_micro _= +1.17; FC_qRT-PCR _= +1.28, p = 0.005; Table 1; Figure 2A and 2B).

To investigate whether changes in *Hif1α *mRNA expression are accompanied by variations at the protein level and to analyse Hif1α localisation in 8.5 dpc mouse embryos, an immunofluorescence analysis was performed on paraffin sections.

**Figure 3:**
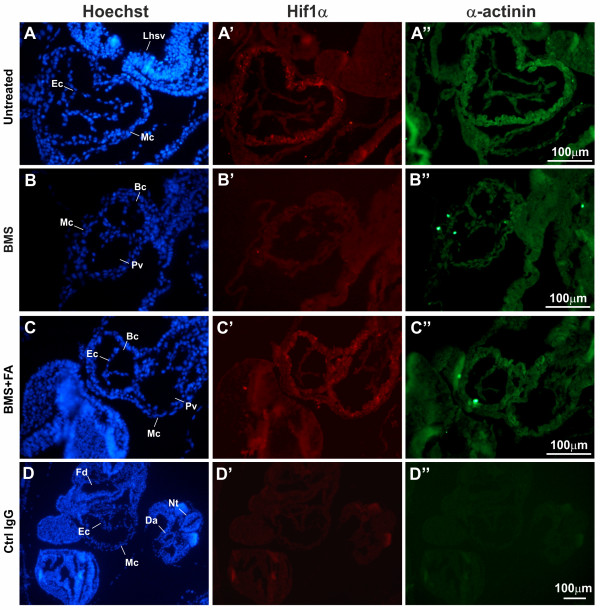
**Immunofluorescence results of Hif1α expression in untreated controls (A'), BMS- (B') and BMS+FA-treated (C') embryos at 8.5dpc**. Transverse sections of untreated controls (A), BMS-treated (B) and BMS+FA-treated (C) embryos at 8.5 dpc were counterstained with Hoechst to show nuclei (blue). Positive Hif1α staining is evident in the myocardium of untreated controls (A') and BMS+FA-treated embryos (C'). Bc = bulbus cordis; Da = dorsal aorta; Ec = endocardium or endocardial tissue; Fd = foregut diverticulum; Lhsv = wall of the left horn of sinus venosus; Mc = myocardium; Nt = neural tube; Pv = primitive ventricle

Hif1α is particularly expressed in the myocardium of untreated controls (Figure 3A') compared to the control gene α-actinin (Figure 3A''), while it is clearly under-expressed in BMS-treated embryos (Figure 3B') compared to the control gene (Figure 3B'') and compared to untreated controls (Figure 3A') and BMS+FA-treated animals (Figure 3C').

### *In silico*-identification of RARE elements in the promoter region of *Hif1α*

There is no evidence in the literature of a specific regulation of the expression of *Hif1α *by retinoic acid. Luo et al. (1997), who cloned and characterised the 2,000 bp region upstream of the ATG of mouse *Hif1α*, described its GC-rich promoter as typical of the so-called housekeeping genes [30].

**Figure 4:**
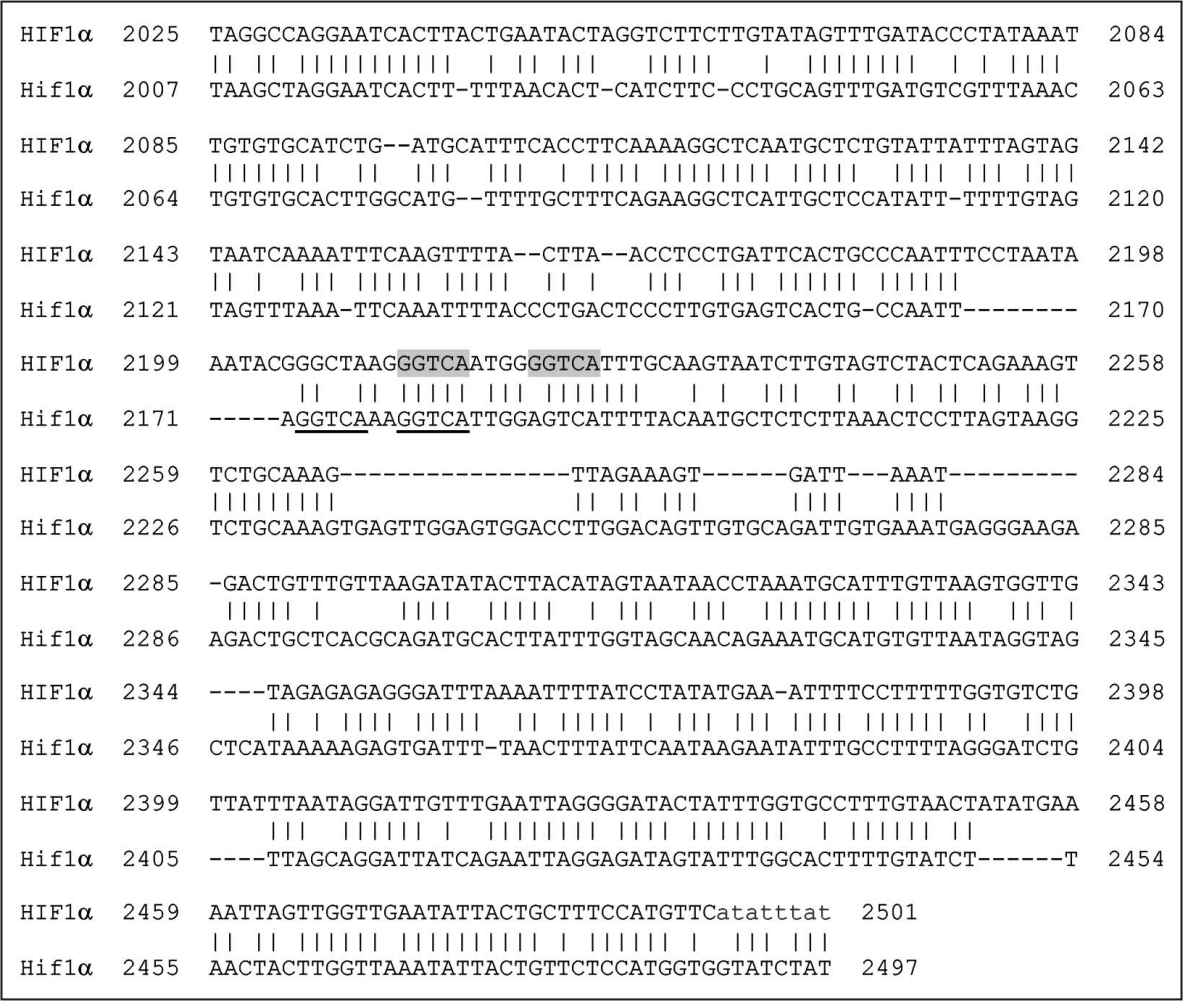
**Alignment of a common 476 bp sequence in the human *HIF1α *(top sequence) and mouse *Hif1α *(bottom sequence) promoter region**. The putative DR2 RARE element in the mouse promoter region is underlined, while the putative DR3 RARE element in the human promoter region is grey coloured.

To verify the presence of putative RAREs far from the ATG, we analysed the aligned mouse and human 5,000 bp sequences. Interestingly, we found numerously sequences with high homology to RAREs. Within a highly conserved region of 476 nt (Figure 4), we found a putative RARE at -2,177nt in the mouse promoter region (Figure 4, bottom sequence). This putative RARE is comprised of a direct repeat (DR) separated by a 2 bp (DR2) element (Figure 4, underlined). In the same highly conserved region of 476 nt, we found a motif resembling a DR3 in the human genomic region (Figure 4, top sequence, in grey).

### Gene expression analysis of Hif1α downstream target genes

*Hif1α *gene encodes the alpha subunit of the heterodimeric transcription factor HIF-1 (Hypoxia Inducible Factor-1), which can promote or repress the transcription of a broad range of genes that are involved in maintaining biological homeostasis.

**Figure 5:**
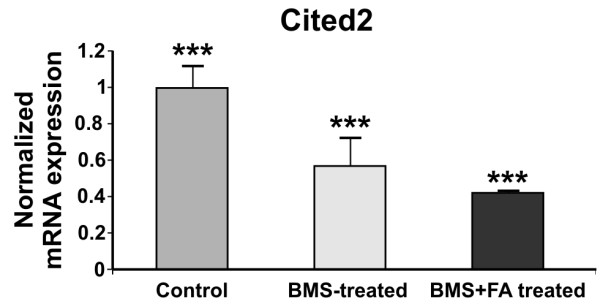
***Cited2 *expression in treated embryos**. Fold change in gene expression analysed by the 2-^ΔΔCt ^(see methods for details) of *Cited2 *in untreated controls, and BMS- and BMS+FA-treated embryos (8.5 dpc). Data are mean ± S.E.M., n = 6; *p < 0.05, **p < 0.01, ***p < 0.0001.

To test whether the observed expression pattern of Hif1*α *in our embryos is correlated with that of a HIF-1 target gene, *Cited2*, we performed qRT-PCR assays. We choose to analyse *Cited2 *for three reasons: 1. it was not present in our microarray slides; 2. it is an important negative regulator of Hif1α; 3. several papers demonstrated its role in cardiac looping and TGA [31]. As expected, *Cited2 *is down-regulated in BMS-treated embryos (FC = -1.94, p < 0.001), but it also remains down-regulated in BMS+FA-treated embryos (FC = -2.36, p < 0.001) (Figure 5).

**Figure 6:**
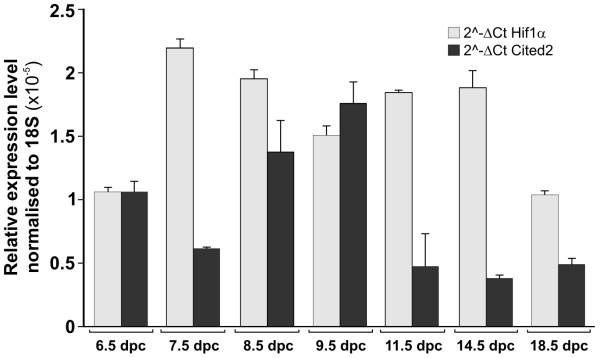
***Hif1α *and *Cited2 *mRNA expression pattern during normal mouse embryogenesis**. Data are mean ± S.E.M., n = 3.

To assess the expression level of *Hif1α *and *Cited2 *during normal mouse development we analysed total RNA isolated from 6.5 to 18.5 dpc mouse embryos by qRT-PCR (Seegene Inc, Korea). Both these genes are widely expressed throughout embryonic development, with a higher level of *Hif1α *compared to *Cited2 *(Figure 6). *Hif1α *shows its highest expression between 7.5 and 8.5 dpc, while for *Cited2*, a peak is observed between 8.5 and 9.5 dpc (Figure 6). Because it is known that the stages from 7.5 to 11.5 dpc are relevant for normal cardiac development, these data further suggest the essential role of a correct dosage of *Hif1α *and its downstream targets, such as *Cited2*, for a normal morphogenesis of the heart.

## Discussion

### Hif1α down-regulation might be responsible for the cardiac defects of mouse embryos

Heart defects are a common feature of congenital human syndromes, as they are present in 25% of all congenital abnormalities, suggesting that genes important in patterning the heart may also have a role in the development of other embryonic structures [1,3,4].

It is well known that a precisely regulated supply of RA is essential for normal cardiogenesis, because both an excess and a deficiency of RA (or vitamin A) have been found to cause teratogenic effects during early heart development [21,32-35].

We previously developed a mouse model for congenital defects, in particular cardiac and thymic abnormalities, by administration to pregnant mice of BMS-189453, an antagonist of retinoic acid that selectively binds to RAR receptors (α, β and γ), and blocks the intake of RA inside the cells. Mice born from pregnant females treated with BMS-189453 showed thymic abnormalities (98%), cardiac defects (81%, 61% of which were D-TGA) and neural tube defects (20%) [21]. These abnormal phenotypes could be partly rescued by oral administration of FA to the pregnant females exposed to BMS-189453 [22].

A comparative analysis of microarray gene expression patterns, identified *Hif1α *as a gene that is down-regulated in BMS-treated embryos and whose expression level is restored (73% vs untreated control) by FA administration. Microarray data were confirmed by qRT-PCR. In parallel, to further support the mRNA results, we used specific antibodies and immunofluorescence analysis, to evaluate Hif1α expression and localisation in mouse embryos at 8.5 dpc. We found that at this embryonic stage Hif1α is expressed in the myocardium (Figure 3A') and that, in line with our microarray and qRT-PCR observations, Hif1α was not detected in sections of BMS-exposed embryos (Figure 3B'), while untreated control (Figure 3A') and BMS+FA-treated embryos (Figure 3C') displayed comparable levels of expression.

Hif1α has an essential role in cardiovascular development, as mice lacking this transcription factor show cardia bifida and cardiac looping defects [36]. Moreover, it has been recently demonstrated that cardiac development in the mouse is characterised by a hypoxic environment with high levels of Hif1α protein [37]. In particular Hif1α is expressed in wild type hearts between E8.0 to E11.5; the highest levels were found at 8.5 and 9.5 dpc, with a decrement at 10.5 that became undetectable by 11.5dpc. In particular, the hypoxic environment in the developing heart initiates a HIF-1 mediated transcriptional program that facilitates the development of a functional heart [37]. These data confirm that Hif1α is not only expressed, but it is functional in the 8.5 dpc developing heart, which is the developmental stage we analysed in our mouse models.

Interestingly, *Cited2*, which is a negative Hif1α regulator and also a HIF-1 inducible gene [38], has an important role in heart development and morphogenesis, in particular, in controlling left-right patterning through a Nodal-Pitx2c pathway [39]. Intriguingly, the human homologue was found to be mutated in patients with D-TGA and heterotaxia [40]. Because *Cited2 *was not present in our microarray, we analysed its mRNA by qRT-PCR. As expected, *Cited2 *is down-regulated in BMS-treated embryos, but it is further down-regulated in BMS+FA-treated embryos (Figure 5). This last result might indicate that when Hif1α expression is raised by supplementation of FA, there is a time window necessary to recover Cited2 expression. This is reasonable because it is known that Cited2 and Hif1α participate to a unique regulatory feedback mechanism to limit excess HIF-1 activation and to maintain normal tissue homeostasis; in this regulatory mechanism, Cited2 dissociates p300 from Hif1α and represses Hif1α activity [41,42]. In line with this hypothesis, we found that the *Hif1α *expression pattern during mouse embryogenesis follows a trend sequentially to *Cited2 *(Figure 6). In fact, *Hif1α *highest expression levels are found at 7.5 and 8.5 dpc, while the highest *Cited2 *levels are at 8.5 and 9.5 dpc (Figure 6).

Finally, while an important role has been well established for HIFα in many human cancers, especially for those that are highly hypoxic [43], and in the pathophysiological responses to hypoxia in pulmonary hypertension and myocardial ischemia [44], the present are the first data linking Hif1α to human CHDs to the best of our knowledge.

### What is the link between *Hif1α *and retinoic acid?

Both excess RA administration and vitamin A deficiency can disturb cardiac looping in embryos [32,33,45,46]. Moreover, it was demonstrated that treatment with retinoic acid induced D-TGA in mice [21,46]. Because it is also well known that *Hif1α *deficiency causes cardiac looping disturbance [36,37,47], it is reasonable to think that a alterations in the metabolism of retinoic acid might influence a correct dosage of *Hif1α*.

However, there is no evidence that RA directly regulates the expression of *Hif1α*, and in fact it was elegantly demonstrated that at E8.5, RA activity is present only in the posterior portion of the heart (atria and inflow tract), whereas Hif1α is expressed throughout the heart [48]. Moreover, in a different system, it was demonstrated that ATRA (all-trans retinoic acid) increases HIF1α protein levels [49]. By microarray and qRT-PCR assays, we found that Hif1α is down-regulated when RA signalling is inhibited; moreover, an *in silico *search performed on the 5,000 bp genomic region upstream the ATG revealed a conserved region of approximately 500 nt in both human and mouse *Hif1α*, containing a putative RARE element resembling a DR2 in the mouse promoter (top sequence, Figure 4). Because it is known that the heterodimer RXR/RAR interacts preferentially with DR2 and DR5 [50]; it would be interesting to verify whether this RARE sequence is functional.

### What is the link between *Hif1α *and folic acid?

Folic acid is an essential vitamin for a wide spectrum of biochemical reactions involved in DNA and RNA metabolism. Impaired folate-dependent metabolism can lead to several pathologies including megaloblastic anaemia, cardiovascular diseases and neural tube and congenital heart defects [51]. While it is well known that maternal supplementation with folic acid during pregnancy lowers the risk of congenital birth defects, the molecular mechanism of action for this phenomenon is still unclear [51,52]. It has recently been shown that in the mouse, maternal folate deficiency significantly affects myocardial cell proliferation with no change in apoptosis levels [53].

Considering that *Hif1α *does not belong to the category of genes involved in FA metabolism, it is difficult to establish what relationship exists with FA. Due to the well-known ability of FA to affect DNA methylation [54], it may be speculated that FA might epigenetically regulate *Hif1α *expression.

## Conclusions

A finely tuned regulation of gene expression during embryogenesis and development is crucial for a normal anatomy and physiology. Though very little is currently known regarding factors that influence and regulate developmental gene expression of the cardiovascular system, emerging large-scale technologies should be of great help to unravel the complex and highly regulated interplay of genes and cell-cell interactions in the developing heart by permitting a complete analysis of embryos transcriptomes [18,20,55].

In this paper, we have identified a discrete number of altered genes that might be involved in congenital defects in the mouse. These defects, in particular CHD and D-TGA, caused by a dysregulation of RA metabolism, were consistently rescued by exogenous administration of folic acid *in vivo*.

Among the altered genes, we have more extensively analysed the expression pattern of *Hif1α*. The down-regulation of Hif1α (both at the mRNA and protein levels) following blocking of retinoic acid intake in the developing mouse, its recovery after oral supplementation with folic acid and its localisation in the cardiac primordia suggest that the observed congenital heart malformation might be due to a de-regulation of Hif1α and its downstream targets (e.g. *Cited2*). Because alteration of both of these genes causes defects in left-right patterning of developing embryos, and the major cardiac defect in our mouse model was D-TGA, the present data support the suggestion to include D-TGA in the group of heterotaxy, a disorder characterised by abnormal lateralisation of normally asymmetric thoracic and abdominal organs.

## Abbreviations

D-TGA: D-transposition of great arteries; FA: folic acid; dpc: days post coitum; FC: fold change; *Hif1α*: hypoxia inducible factor 1 alpha subunit; BMS-treated: BMS189453 treatment; BMS+FA-treated: BMS189453 treatment plus folic acid supplementation; RARE: retinoic acid responsive elements.

## Authors' contributions

FA designed most of the experiments, coordinated the study and wrote the manuscript. LD performed microarray experiments and participated in the interpretation of the results and in writing part of the manuscript. LC carried out immunofluorescence experiments and participated in the interpretation of the results and in writing part of the manuscript. LV carried out qRT-PCRs and participated to the interpretation of the results. DC performed mouse treatments. SB, GP, GP, AD analysed microarray raw data, interpreted the results and wrote part of the manuscript. GS participated in the interpretation of immunofluorescence results and revised the manuscript. BM and GN together contributed to coordinate the study and edited the manuscript. All authors read and approved the final manuscript.

## Supplementary Material

Additional file 1 Table S1: List of the 477 differentially expressed genes (FC≥ ± 1.5) in BMS189453-treated embryos Click here for file

Additional file 2 Table S2: List of the 239 differentially expressed genes (FC≥ ± 1.5) in BMS+FA-treated embryos Click here for file
